# Light-Driven Synthetic Biology: Progress in Research and Industrialization of Cyanobacterial Cell Factory

**DOI:** 10.3390/life12101537

**Published:** 2022-10-03

**Authors:** Chaofeng Li, Jiyang Zheng, Yushuang Wu, Xiaotong Wang, Hui Shao, Dong Yan

**Affiliations:** Shanghai Lumy Biotechnology Co., Ltd., Shanghai 201100, China

**Keywords:** synthetic biology, cyanobacteria, biosynthesis, metabolic engineering, CO_2_ utilization

## Abstract

Light-driven synthetic biology refers to an autotrophic microorganisms-based research platform that remodels microbial metabolism through synthetic biology and directly converts light energy into bio-based chemicals. This technology can help achieve the goal of carbon neutrality while promoting green production. Cyanobacteria are photosynthetic microorganisms that use light and CO_2_ for growth and production. They thus possess unique advantages as “autotrophic cell factories”. Various fuels and chemicals have been synthesized by cyanobacteria, indicating their important roles in research and industrial application. This review summarized the progresses and remaining challenges in light-driven cyanobacterial cell factory. The choice of chassis cells, strategies used in metabolic engineering, and the methods for high-value CO_2_ utilization will be discussed.

## 1. Introduction

Industrial biotechnology led by synthetic biology has become the strategic commanding height of economic development. In addition, faced with various challenges including global resource shortage and environmental pollution, synthetic biotechnology provides new ideas for the sustainable development through natural or artificial design, transformation, and creation of microbial cell factories [1,2].

At present, the commonly used chassis microorganisms in synthetic biology, such as *Escherichia coli*, *Saccharomyces cerevisiae*, *Bacillus subtilis* and *Corynebacterium glutamicum*, have a heavy dependence on organic carbon sources such as glucose in the production of target compounds [3]. Carbon recycling is one of the crowning achievements of synthetic biology. Nevertheless, the growth and production of heterotrophic microorganisms consume organic carbon, making the whole process carbon dioxide (CO_2_)-emitting. Therefore, a new generation of synthetic biology platforms is needed to deal with the urgent problems in the traditional synthetic biotechnology, including the large dependency of organic carbon sources, the high cost of substrates, and net carbon emission [4].

**Figure 1:**
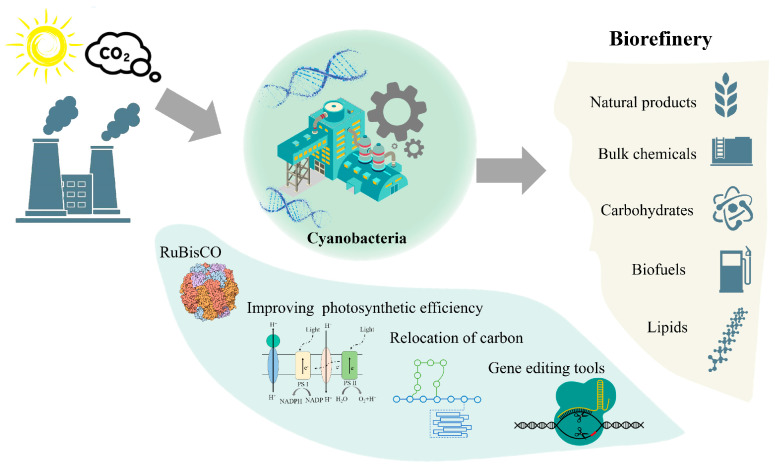
Schematic diagram of synthetic biomanufacturing in light-driven cell factories and relative metabolic engineering strategies.

Oxygenic photosynthetic microorganisms have been living on the earth for more than 2 billion years. They photosynthesize and release oxygen when they grow in the light. They can use solar energy as the sole energy and CO_2_ as the sole carbon source for growth [5]. Those photoautotrophic microorganisms are usually used as chassis organisms in light-driven synthetic biology, which directly convert CO_2_ into target products in a carbon-negative mode. This CO_2_ utilization technology can promote the realization of “carbon neutrality”. Cyanobacteria are prokaryotic oxygenic photosynthetic microorganisms. They have become potential photosynthetic chassis microorganisms and are also known as “green *E. coli*”. They have many advantages, including the small genome, simple genetic background, low nutrient requirement, and high CO_2_ fixation efficiency [6]. In recent years, cyanobacteria have been used as light-driven cell factories in the production of a number of products of bioenergy and bio-based chemicals (Figure 1). They also play increasingly important roles in medicine and healthcare, environmental protection, and agriculture [7,8,9]. In addition, eukaryotic photosynthetic microorganisms are also an optional chassis for light-driven synthetic biology, although it is beyond the scope of this review.

This paper focuses on the development of cyanobacterial cell factories and systematically summarizes the chassis selection of cyanobacterial species, the tools in the construction of light-driven cell factories, and the methods for high-value CO_2_ utilization. We also reviewed the overall challenges in cyanobacterial cell factories, including the low fitness of standardized modules, genetic instability of the engineered cells, and the problems in scale-up processes.

## 2. Chassis Selection of Engineered Cyanobacteria

Cyanobacteria are one of the oldest living organisms on earth. They have a great variety and a wide distribution—they are found in the seawater, freshwater, hot springs, and various extreme environments. Multi-cellular forms have been found in cyanobacteria, including unicellular, filamentous, and undifferentiated filaments. Different cyanobacteria may thus differ in the physiological metabolisms, such as photosynthetic-carbon-fixation efficiency, carbon sink pathways, and nitrogen fixation capacity [10,11]. Considering the difficulty of the metabolic transformation and future engineering applications, the reasonable selection of chassis cells is of great significance in order to construct efficient and stable photosynthetic cell factories [6]. The commonly used model algal species include *Synechocystis* sp. PCC6803, *Synechococcus elongatus* PCC7942, *Anabaena* sp. PCC7120, *Synechococcus elongatus* UTEX2973, and *Synechococcus* sp. PCC7002.

*Synechocystis* sp. PCC6803 was collected from freshwater. With a genome size of 3.6 Mbp, it is the first cyanobacterial species to be sequenced. Like most cyanobacteria, DNA transfer of PCC6803 is usually achieved via natural transformation with pre-designed homology regions at the neutral sites [12]. DNA transfer can also be achieved by cell conjugation, ultrasonic transformation, and electroporation [13,14]. *Synechocystis* sp. PCC6803 is a viable platform for studying photosynthesis, though the growth rate was slower than that of *S. elongatus* PCC7942 and *S. elongatus* UTEX2973 [15]. The studies on the photosynthetic production of ethanol were mainly from this strain. In *Synechocystis* sp. PCC6803, the pyruvate decarboxylase derived from *Zymomonas mobilis* was expressed and the promoter strength was optimized. Meanwhile, the endogenous alcohol dehydrogenase of *Synechocystis* sp. PCC6803 was overexpressed. By impeding the biosynthesis of β-hydroxybutyrate, the yield of ethanol increased from 0.5 g L^−1^ to 5.5 g L^−1^ [16,17]. In addition, *Synechocystis* sp. PCC6803 was also used to produce lactic acid [18], free fatty acids and antibiotics [19], ethylene [20], astaxanthin [21], aromatic amino acids and phenylpropanoids [22].

As a freshwater unicellular photosynthetic bacterium, *S.* *elongatus* PCC7942 has a long history in genetic engineering and is one of the most well-studied species in photosynthesis and genetic manipulation. It has a genome size of around 2.7 Mbp. Natural transformation is the common way of DNA transfer for this strain, while methods of cell conjugation and electroporation have also been reported [23]. Deng et al. introduced the pyruvate decarboxylase gene *pdc* and the alcohol dehydrogenase II gene *adhII* from *Z. mobilis* into *S.* *elongatus* PCC7942 via the plasmid pCB4. The expression of the genes was controlled by the rbc promoter. The yield of ethanol reached 0.23 g L^−1^. This is the first report of biofuel synthesis in genetic engineered cyanobacteria [24]. *S.* *elongatus* PCC7942 was also used in the light-driven biosynthesis of various chemicals, including n-butanol [25], isobutanol [26], 2,3-butanediol [27], sucrose [28], isopropanol [29] and acetone [30].

*Anabaena* sp. PCC7120, also known as *Nostoc* sp. PCC7120, is a freshwater multicellular filamentous cyanobacterium [31] with a genome size of approximately 6.4 Mbp. *Anabaena* sp. PCC7120 is a model species in the study of cell differentiation. Cell conjugation is usually used for DNA transfer for this strain, while electroporation was also reported [32,33,34]. *Anabaena* sp. PCC7120 is characterized by specialized cells called heterocysts wherein N_2_ can be efficiently reduced and fixed. This feature can be used to produce hydrogen [35]. A genome-scale study investigating the metabolisms of the two cell types in *Anabaena* sp. PCC7120, vegetative cells and heterocyst, found that the exchange of at least four metabolites was necessary for the optimal growth under nitrogen-replete and -deplete conditions [36]. The study gave us a better understanding of the metabolic network of *Anabaena* sp. PCC7120 and possibly enabled a more efficient engineering of nitrogen fixing multicellular cyanobacteria.

*S.elongatus* UTEX2973 is a newly discovered algal strain with good tolerance to high temperature and strong light conditions. It has a fast growth rate with a generation time of only 1.9 h and has a huge potential for biological production. The genome size of *S.* *elongatus* UTEX2973 is 2.7 Mbp, and triparental mating is the common form of DNA transfer [15]. Li et al. introduced the *pilN* gene into *S.* *elongatus* UTEX2973 in order to make the strain naturally transformable [37]. The accumulation of glycogen and sucrose was investigated in this strain. The content of the accumulated glycogen reached 51% of the dry cell weight under nitrogen-deplete conditions. In addition, over 94% of cellular produced sucrose was excreted with the sucrose-proton permease CscB under salt stress. The produced sucrose reached 8.7 g L^−1^ in a 21-day semi-continuous culture [38]. In 2018, The transcriptional start site in the whole genome of *S.* *elongatus* UTEX2973 was accurately identified, providing the basis to study the transcriptional control and metabolic engineering of the strain [39].

*Synechococcus* sp. PCC7002 is a marine cyanobacterium with a genome size of 3.01 Mbp. Natural transformation and cell conjugation are commonly used for DNA transfer in this strain [40,41]. Compared with freshwater photosynthetic bacteria, *Synechococcus* sp. PCC7002 has many advantages: it has a high optimum growth temperature of 38 °C; it can tolerate a continuous light intensity of 500 μmol photons m^−2^ s^−1^, while the optimum light intensity for *Synechocystis* sp. PCC6803 and *S.* *elongatus* PCC7942 is 300 μmol photons m^−2^ s^−1^; it has a wide salt tolerance, so seawater can be directly used for large-scale cultivation. Those features provide it significant advantages for industrial use [42]. *S.* *elongatus* PCC7002 has been used to produce various chemicals such as: hydrogen fuel [43], lysine [44], lactic acid, 2,3-butanediol [45], mannitol [46], limonene [47], free fatty acids [48] and polymers [49].

In addition to the above model algal species, Włodarczyk et al. recently reported a new strain of *Synechococcus* sp. PCC11901. PCC11901 can achieve DNA transfer through natural transformation. The doubling time was around 2 h, less than that of yeast. The accumulated biomass of cell dry weight reached up to 33 g L^−1^. The yield of free fatty acids produced by PCC11901 was over 1.5 g L^−1^, which is comparable to that of gene-modified heterotrophic microorganisms [50]. The results indicate that PCC11901 is a promising candidate for a photosynthetic cell factory. A suitable chassis could be selected according to the target products, as well as the metabolic transformation potential of the strain (Table 1).

## 3. Enabling Technologies for Light-Driven Synthetic Biology

**Figure 2:**
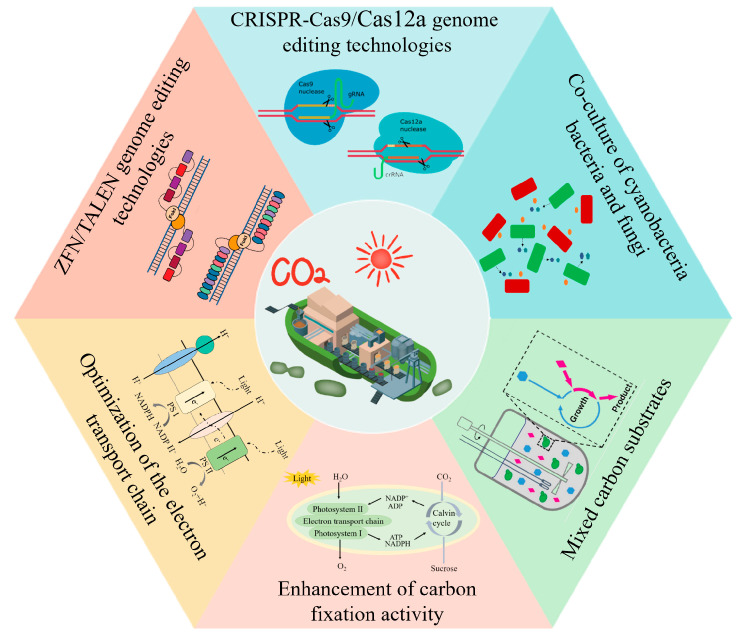
Synthetic biology provides a wealth of enabling tools for the development of light-driven cell factories.

Synthetic biology offers a wealth of useful tools for light-driven cyanobacterial cell factories. In the engineering of cell factories, ZFN, TALEN, and RNA-mediated CRISPR-Cas systems have been applied to target product production. In the field of light-driven synthetic biology, increased efficiency in light utilization and carbon fixation encourages the realization of the full potential of carbon-negative manufacturing. In addition, the construction of an “autotrophic–heterotrophic” symbiotic system has been a hot topic in recent years because it provides ways for the direct transformation of inorganic carbon (CO_2_) into some major chemical products and high-value products (Figure 2).

### 3.1. Gene-Editing Technology

#### 3.1.1. ZFNs and TALENs

Synthetic biology provides rich enabling tools for the light-driven cell factories (Figure 2). The main purpose of light-driven synthetic biology is to modify or reconstruct microbial cells to endow them with specific physiological functions to produce target products. Therefore, developing gene manipulation tools that are convenient, efficient, and precise is an important part of the research and development. With the development of novel genetic tools, zinc finger nucleases (ZFNs), transcription activator-responsive nucleases (TALENs), and clustered regularly interspaced short palindromic repeats and related proteins (CRISPR-Cas9) technologies have been used in a broad range of fields in the synthetic biological fields, including biology, medical research, and metabolic engineering [52,53,54,55].

ZFNs and TALENs were widely used gene-editing tools in the past 20 years [56,57]. However, not many successful gene-editing cases using those methods have been published. The successful use of ZFN gene editing in *Chlamydomonas* has been reported [58]. The zinc finger domain consisted of 3–6 nucleotide triplets. Since the nucleases to which they are attached can only function as dimers, creating effective gene-editing strategies is still challenging despite the weak off-target effects. For TALENs, each domain recognizes a single nucleotide instead of DNA triplet, suggesting the technology could modify most sequences. The construction of each nuclease needs to be carefully designed, and this technique is susceptible to cytosine methylation [59].

#### 3.1.2. CRISPR-Cas9 and CRISPR-Cas12a

At present, the most advanced technology for cyanobacterial genetic engineering is the CRISPR-Cas system. It is efficient due to the simple target design, efficient working system, and multi-target gene editing. Therefore, it has become the third-generation gene-editing technology after ZFN and TALENs [60]. This technology is derived from the bacterial antiviral defense system. The core components consisted of guide RNA (sgRNA) and Cas9 protein. The design of sgRNA requires complete genomic data to prevent a high off-target effect. The Cas endonuclease assembles with a guide RNA sequence of around 20 bp, which is complementary to the target DNA by the principle of base pairing. The complex then leads to a double-strand break (DSB) at the target site. DSBs can be repaired by DNA repair mechanisms through non-homologous end joining, or if a suitable template is available, homology-directed repair.

Gene editing with the CRISPR-Cas9 system enhances the rate of homologous recombination, thereby greatly improving the efficiency of cyanobacterial genome editing. This has been demonstrated using *S.* *elongatus* PCC7942, for which a 57% increase in recombination efficiency was found [61]. Li et al. applied the CRISPR-Cas9 technology to the succinate production in cyanobacteria. The *glgC* gene that encodes a glucose-1-phosphate adenosyltransferase necessary for glycogen synthesis was knocked out. The succinate titer of the recombinant strain reached 0.18 mg L^−1^. The *ppc* and *gltA* genes at the *glgC* gene locus were then knocked in using the CRISPR-Cas9 tool to improve the tricarboxylic acid cycle, which further increased the titer of succinate to 0.44 mg L^−1^. The results suggested that the cyanobacterial metabolic engineering based on the CRISPR-Cas9 system was feasible [61]. However, Wendt and Li et al. found that high-level expression of Cas9 decreased the cell viability of *S.* *elongatus* UTEX2973 and *S.* *elongatus* PCC7942 [62]. A new strategy based on CRISPR using the CRISPR-associated protein Cas12a (previously known as Cpf1) was thus applied in genome editing and DNA assembly to reduce the toxicity to cyanobacteria cells. The CRISPR-associated protein Cas12a is an endonuclease from the type V-A CRISPR system. This technology does not produce toxicity, and the editing becomes simpler because only CRISPR-RNA (crDNA) is required. After Cas12 recognizes the spacer-adjacent motif (PAM) sequence, it targets 17 nucleotides downstream of 5′-YTN-3′ to cut double-stranded DNA (dsDNA) in a 5′-end staggered shearing manner under the guidance of mature crDNA [63]. Compared to Cas9, Cas12a does not require the transactivation of crRNAs and provides more precise genome editing since it separates dsDNA from PAM sequences, making CRISPR-Cas12a a versatile tool for genome editing. The reported success rate reached 97% in the gene editing of *S.* *elongatus* PCC7942 using this tool [64].

### 3.2. Enhancement of Photosynthetic Efficiency

Cyanobacteria are generally considered to be one of the most promising photosynthetic microbial chassis. The light utilization efficiency of cyanobacteria is generally 3–9% dozens of times higher than that of terrestrial plants, and there is still room for improvement. Continuous efforts have been made to improve photosynthetic efficiency [65,66]. For example, the *apcE* gene in *Synechocystis* PCC6803 was knocked out, so phycobilisomes could not be fixed on thylakoid membranes. This resulted in a 60% increase in growth rate due to the reduced light-harvesting density and higher light-harvesting efficiency [67]. Another strategy is to increase the ratio of the produced ATP to NADPH via the photosynthetic electron transport chain. Generally, the produced ATP is only 1.28 times more than NADPH, while the ATP/NADPH ratio that is needed in carbon fixation is 1.5, suggesting ATP shortage. Akihiko et al. reported that the overexpression of the *flv3* gene that encodes the flavodiiron protein involved in alternative electron flow associated with O_2_ photoreduction via NADPH in *Synechocystis* PCC6803 increased the supply ratio of ATP/NADPH and significantly improved the biomass and growth rate of the strain [68].

In 2010, Chen et al. identified a new type of chlorophyll, chlorophyll *f*, that can absorb red and infrared light [69]. This could be used to broaden the range of the light absorption spectrum of photosynthetic organisms. In 2018, Nurnberg et al. found that a cyanobacterium, *Chroococcidiopsis thermalis*, can switch the light energy utilization mode between the infrared and the visible light owing to the possession of chlorophyll *f* [70]. This discovery broadens the traditional understanding of photosynthesis and provides new research ideas for the light utilization to avoid photodamage. In addition, the mass production of target products in engineered cyanobacteria improves photosynthetic efficiency. For example, Ducat et al. [28] modified *S.* *elongatus* PCC7942 to produce sucrose. The results showed that the activity of Photosystem II (PSII), the chlorophyll content, and carbon-fixation capacity increased. Increased photosynthetic carbon-fixation rate was also observed in engineered bacteria that produced ethanol and isoprene [17].

### 3.3. Enhancement of Carbon-Fixation Efficiency

Improving the efficiency of carbon fixation and carbon conversion of cyanobacterial chassis strains enhances the utilization efficiency of the captured light and thus improves the light-driven carbon-fixing cell factory. Five aspects are usually considered to achieve the goal: (1) increase the efficiency of the carbon absorption and carbon concentrating mechanisms; (2) increase the carbon-fixation efficiency; (3) develop new carbon-fixation pathways; (4) reduce inorganic carbon loss; (5) expand the scope of carbon sources [32,71,72]. In order to improve the carbon-fixation efficiency of cyanobacteria, the RuBisCO gene from *Synechococcus elongatus* PCC6301 was overexpressed in an isobutyraldehyde-synthesizing strain of *S.* *elongatus* PCC7942. This resulted in an increased yield of the target product. In addition, the non-oxidative glycolysis pathway in *E. coli* was redesigned that one molecule of glucose was broken into three molecules of acetyl-CoA to avoid the loss of the two carbon units in glycolysis. The theoretical value of the maximum carbon conversion efficiency was thus increased [73]. The same concept was applied to the genetic engineering of cyanobacteria that the “malonyl-CoA-glycerate” pathway was designed for the remodeling of the metabolic network of *S.* *elongatus* PCC7942. In the new pathway, a molecule of a three-carbon substrate (phosphoenolpyruvate) was combined with one molecule of CO_2_ to generate glyoxylic acid that was then converted to two molecules of acetyl-CoA, thereby avoiding carbon loss and increased the concentration of intracellular CoA [74]. The core of developing cyanobacterial carbon-negative cell factories is to extend and expand the carbon flow of cyanobacteria by introducing exogenous pathways or modifying existing natural metabolic pathways. This means more carbon from the Calvin–Benson cycle need to be directed to target pathways. The improvement of the overall carbon-fixation efficiency through the effective control of input and output of carbon flow thus becomes possible.

### 3.4. “Autotrophic–Heterotrophic” Symbiosis Platform

The co-culturing system of cyanobacteria and heterotrophic microorganisms in order to build “autotrophic–heterotrophic” symbiotic platforms has become a research hotspot in recent years. In the system, photosynthetic microorganisms use light energy to synthesize organic matters that are necessary for themselves and their partners to meet the needs of heterotrophic microorganisms. On the other hand, heterotrophic microorganisms provide inorganic nutrients and other growth factors to photosynthetic microorganisms [75,76]. The strains in the mixed bacterial system are thus interdependent, which provides the co-culture system some advantages compared with pure culture: the robustness of the culture system is enhanced [77]; the utilization efficiency of substrates in production processes is improved [78]; the production capacity of high value-added biochemical products are increased; the costs of carbon sources are saved [79]. The co-culture system plays important roles in environmental governance and microbial cell factories and has a broad range of applications, e.g., sewage treatment [80], soil remediation [81], biodegradation [82,83], fatty acids production [84], sugar production [85], fuel production [86,87], etc.

Using the organic carbon synthesized by cyanobacteria to support the production of biochemicals of heterotrophic microorganisms is a promising direction in the construction of co-culture systems with artificial photosynthetic microorganisms. For example, the introduction of the sucrose-proton permease CscB into *S.* *elongatus* PCC7942 resulted in a sucrose yield of 28 mg L^−1^ h^−1^ of the modified strain. The modified strain was used in the co-culture with *Halomonas boliviensis*, *E.coli*, and *B. subtilis* in “autotrophic–heterotrophic” symbiosis platforms to produce some high-value-added products such as polyhydroxybutyric acid and amylase [28,77,88]. A cross-feeding symbiosis system was built by the co-culturing of a sugar-producing strain of *S. elongatus* PCC7942 and a diazotroph *Azotobacter vinelandii*. *S.* *elongatus* PCC7942 provided carbon, while *A. vinelandii* provided nitrogen to the system. The results showed that the production of industrially relevant products without adding any additional organic carbon source and nitrogen source was possible [89]. Zhang et al. constructed an artificial autotrophic–heterotrophic symbiosis system by co-culturing a sucrose-producing strain of *S. elongatus* UTEX2973 and a 3-hydroxypropionic acid-producing strain of *E. coli* [90]. Zuñiga et al. built four community metabolism models in which a sucrose-secreting strain of cyanobacteria was co-cultured with *E. coli* K-12, *E. coli* W, *Yarrowia lipolytica*, and *B. subtilis*, respectively. Metabolic exchanges between the autotrophic and each heterotrophic partner were finally maintained in the matrix with the lowest organic carbon supply [91].

## 4. Research Progresses in the Application of Light-Driven Cyanobacterial Cell Factory

As a carbon-negative cell factory, cyanobacteria can directly convert light energy and CO_2_ into biofuels and bio-based chemicals through the photosynthetic carbon fixation. This is a brand-new bio-manufacturing mode. At present, the growth rates of various *Synechococcus* chassis have exceeded that of heterotrophic microorganism chassis such as yeast, which uses glucose as substrates, and the reported OD_730_ value reached more than 200 [50]. Meanwhile, the yield of light-driven synthesis of various compounds has reached above the gram level, and some even exceeded that of heterotrophic cell factories using *E. coli*. Generally, products synthesized in heterotrophic microorganisms and natural products can both be synthesized in photosynthetic microorganisms directly from CO_2_. This includes biofuels, bulk chemicals, degradable plastics, sugars, and plant-originated natural products. Particularly, in the synthesis of high-value natural products, cyanobacteria have unique advantages such as the possession of the thylakoid membrane system and the photosynthetic electron transport chain, which allows the efficient synthesis of target products. It is thus expected to subvert the traditional industrial chain in a greener way.

### 4.1. Biofuels

Ethanol is the first biofuel product that achieved commercial promotion and application. Its application as a fuel additive and substitute has been widely accepted. Currently, most of the bioethanol comes from the biorefinery process. Velmurugan et al. knocked out the *glgC* gene encoding the glucose-1-phosphate adenosyltransferase in the glycogen synthesis pathway and introduced the pyruvate decarboxylase gene *pdc* from yeast and the alcohol dehydrogenase *adh* gene from cyanobacteria. The ethanol production reached 3856 mg L^−1^ when cofactors of Mg^2+^, Zn^2+^, thiamine pyrophosphate, and NADP^+^ were provided [92].

Hydrogen, one of the most promising clean fuels, releases only water and produces a high amount of heat per mole when combusted with oxygen. At present, the biological hydrogen production is mainly conducted with three biological groups: bacteria, green algae, and cyanobacteria. Hydrogen production was proved to be more successful in cyanobacteria owing to the excellent transformation efficiency and the relatively clear genetic background. On the other hand, the use of genetically engineered eukaryotic microalgae for hydrogen production was rarely reported. Filamentous cyanobacteria are preferred for hydrogen production due to the natural anaerobic environment of heterocysts. Three enzymes are involved in hydrogen metabolism in *Anabaena* sp. PCC7120: nitrogenase, bidirectional hydrogenase (Hox), and hydrogenase (Hup). The mutants of Δ*hupL* and Δ*hupL*/Δ*hoxH* both produce 3–6 times higher hydrogen than the wild type [93]. Other biofuels have also been produced in cyanobacteria. For example, the production pathways of isobutyraldehyde and isobutanol were constructed in *S. elongatus* PCC7942 with RuBisCO overexpression. The strain SA597 produced 459 mg L^−1^ of isobutanol in 6 days, and the yield of isobutyraldehyde was 6230 µg L^−1^ h^−1^ [26].

### 4.2. Bulk Chemicals

**Figure 3:**
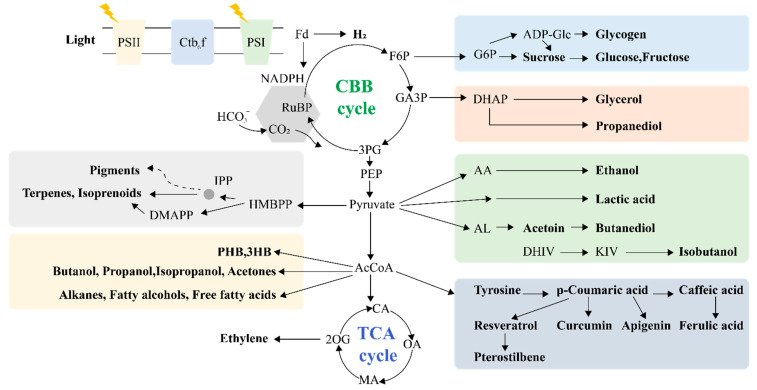
Carbon flux through the metabolic branching points.

Cyanobacteria can also be used to produce diols such as 2,3-butanediol. Starting from pyruvate, the reactions occur step by step by adding acetolactate synthase, acetolactate decarboxylase, and alcohol dehydrogenase. Acetolactate synthase catalyzes the condensation of two pyruvate molecules into one acetolactate, which is then decarboxylated to acetylacetone by acetolactate decarboxylase. The final step is to reduce acetylacetone to 2,3-butanediol with alcohol dehydrogenase (Figure 3). Using this method, the yield of 2,3-butanediol reached 2.4 g L^−1^ in modified *S. elongatus* PCC7942 after 20 days of culturing [27]. In modified *Synechocystis* sp. PCC6803, 2,3-butanediol reached a final titer of 430 mg L^−1^ [94]. Liu et al. constructed an oxygen-sensitive 1,3-propanediol biosynthesis pathway in the heterocyst of *Anabaena* sp. PCC7120, and the incompatibility of oxygen-sensitive enzymes was thus solved by using the regionalization of different reactions. The recombinant strain P11 accumulated 46 mg L^−1^ of 1,3-propanediol, which was 1.7 times that of the control strains without heterocyst [95].

Lactic acid is a chiral *α*-hydroxy acid that is widely used in cosmetics, medicine, chemical industry, textile, food, and other industries. A multimeric form of lactic acid, polylactic acid, is biodegradable and is becoming an alternative to bio-based plastics. There are two isomers of lactic acid, D type and L type. In cells, pyruvate is converted to lactic acid isomers through the corresponding dehydrogenase. In *Synechocystis* sp. PCC6803, the L-lactic acid production was 1.8 g L^−1^, much higher than that of D-lactic acid due to the lower activity of D-lactic acid dehydrogenase [18,96,97]. An efficient light-driven system for producing D-lactic acid from CO_2_ was constructed using *S. elongatus* PCC7942. The D-lactate dehydrogenase from *Lactobacillus bulgaricus* ATCC11842 was mutated, and the cofactor was altered from NADH to NADPH. This increased the production of lactic acid by more than 3.6 times [98].

In a genetically engineered strain of *S. elongatus* PCC7942, YW1, glycerol-3-phosphatase was overexpressed, resulting in a glycerol yield of 1.17 g L^−1^, suggesting that C3 chemicals can be directly produced through photosynthetic production through CO_2_. The pathways of the YW1 strain were further expanded and dihydroxyacetone and 3-hydroxypropionic acid were produced. The results indicated that glycerol produced by the strain itself could be used as a fermentation feedstock [99].

### 4.3. Carbohydrates

Cyanobacteria accumulate sucrose under salt stress. Ducat et al. introduced the *cscB* gene from *E. coli* into *S. elongatus* PCC7942, which was the first report of sucrose excretion from cells of cyanobacteria [28]. In *E. coli* cells, CscB protein is a sucrose permease, which aids the absorption of proton/sucrose under acidic conditions. Since the culturing conditions of cyanobacteria are usually alkaline, cyanobacteria cells simultaneously pump out sucrose and protons synthesized under salt stress. Under the salt stress of 150 mM NaCl, the sucrose production rate of the recombinant strain of *S. elongatus* PCC7942 was 28 mg L^−1^ h^−1^. The sucrose yield reached 2.7 g L^−1^ after 168 h of cultivation under salt stress [28]. The same method was applied to *S. elongatus* UTEX2973, which produced 8 g L^−1^ of sucrose under salt stress [100]. The accumulated glycogen reached 51% of the dry cell weight under nitrogen-deplete conditions of the gene-modified *S. elongatus* UTEX2973. Under salt stress, the strain excreted over 94% of cellular produced sucrose when gene *CscB* was introduced [38].

The secretion of glucose and fructose in *S. elongatus* PCC7942 was achieved by overexpressing the invertases *InvA*, which encodes the enzyme that converts sucrose to glucose and fructose, and *glf*, which encodes a sugar transport factor [96]. The overexpression of the three genes *invA*, *glf*, and *galU* in *S. elongatus* PCC7942 resulted in a total hexose production of 45 mg L^−1^ under the salt stress of 200 mM NaCl [101]. Fan et al. introduced the xylose transporter, Ec-XylE, from *E. coli* and the NADPH-dependent xylose reductase, Cb-XR, from *Candida boidinin* to *S. elongatus* PCC7942. The maximum xylose productivity reached 0.15 g L^−1^ day^−1^ OD^−1^, with an average yield of 0.9 g g^−1^ [102].

### 4.4. High-Value Natural Products

Cyanobacteria have become an ideal platform to produce plant-originated natural products because they have better adaptability to the plant-originated enzyme, better accessibility to reducing power, and less dependency on expensive substrates, which are common problems faced by traditional biological methods. Natural products such as terpenoids and phenylpropanoids have already been produced using cyanobacteria. Ni et al. constructed a photoautotrophic synthesis platform in *S. elongatus* PCC7942 to produce resveratrol, naringenin, bisdemethoxycurcumin, *p*-coumaric acid, caffeic acid, and ferulic acid. The products can be further converted to some other precious and extremely valuable natural products [103]. The same group created a metabolic trap to redirect more than 30% of the source carbon to the low-flow shikimate pathway by introducing the 2-phenylethanol pathway and an artificial de-feedback inhibitory module for the efficient synthesis of natural aromatics products directly from CO_2_ [104].

Isoprene is an important chemical raw material that can be used to synthesize rubber, plastics, and terpenoids by forming polymers [105]. Gao et al. introduced isoprene synthase genes (*ispS*) from different sources into *S. elongatus* PCC7942 and found that the *ispS* derived from *Eucalyptus globulus* was more soluble in cyanobacteria than that from other sources. Subsequently, the bottleneck of the MEP pathway was found by dynamic metabolic flux analysis. The isoprene pyrophosphate isomerase (IDI) and *IspS* fusion enzyme were introduced, and the genes that encode 1-deoxy-d-xylulose 5-phosphate synthase (*dxs*) and 4-hydroxy-3-methyl-2-enyl diphosphate synthase (*ispG*) were highly expressed. The recombinant strain of *S. elongatus* PCC7942 produced 1.26 g L^−1^ isoprene within 21 h [106]. In addition, Brey et al. expressed *E. coli*-derived de-feedback inhibitory enzyme genes, *AroG* and *TyrA*, in *Synechocystis* sp. PCC6803 to produce the precursors of phenylpropanoids, phenylalanine and tyrosine, from CO_2_. The titers reached 580 mg L^−1^ and 41 mg L^−1^, respectively. The production of these two amino acids was estimated to account for 56% of the total fixed carbon. Functions of several enzymes involved in phenylpropanoid biosynthesis were further tested in *Synechocystis* sp. PCC6803, and 207 mg L^−1^ of *p*-coumaric acid, 114 mg L^−1^ of cinnamic acid, and 12.6 mg L^−1^ of caffeic acid were obtained after 6 days of photoautotrophic growth [22].

## 5. The Industrialization of Light-Driven Cyanobacterial Cell Factory

The relative simplicity of cyanobacteria makes it the most promising and most often used chassis organisms in light-driven synthetic biology. The same is true in industrial applications.

Biofuels represent a great attempt at the commercialization of light-driven synthetic biology. Many pioneering companies, such as Algenol Biofuels Inc. (Fort Myers, FL, USA), Joule Unlimited Technologies Inc. (Bedford, MA 01730, USA), and Sapphire Energy Inc. (San Diego, CA, USA), attempted to commercialize biofuels, though they ultimately failed. Among those efforts, Joule completed a 2-year pilot testing of diesel and ethanol production, trademarked Sunflow-D and Sunflow-E, respectively. They received approval from the US Environmental Protection Agency (EPA) for the Sunflow-E as an advanced biofuel in 2016 [107]. At present, Cemvita Factory Inc. (Houston, TX, USA) remains committed to sustainable aviation fuels (SAF) using cyanobacteria, and United Airlines recently announced a new partnership with them in this direction.

Bulk chemicals are the fastest-growing application field for the commercialization of light-driven synthetic biology. Company Photanol B.V. (Amsterdam, Noord-Holland, Netherlands) is the most representative company in the field of application. Based on synthetic biology using cyanobacteria as the chassis, Photanol has built pipelines of bulk chemicals including L-lactic acid [108], 1,3-propanediol [109], and glycolic acid [110]. At present, the production capacity of the bulk chemicals has reached 10 tons year^−1^. Photanol plans to expand it to 30,000 tons per year^−1^ next year and launch its products to the market in 2024.

Due to the unique characteristics of microalgae, high-value-added products, especially plant-originated natural products, are potential target products for the industrial application of light-driven synthetic biology. Some companies, such as Algae-C, Inc. (Caledon, ON, Canada), Provectus Algae, Pty Ltd. (Noosaville, Queensland, Australia), and Lumy Bio Co., Ltd. (Shanghai, China) have emerged, though the application field started late. Algae-C is the fastest-growing company in the field, with patents showing cannabinoids being the main product of the pipeline [111]. In 2021, Algae-C announced that it would expand its production scale with Arizona Algae. The production has begun in the fall of 2021.

Finally, what is worth mentioning is the industrialized application of light-driven synthetic biology in protein production. Jester et al. from Lumen Bioscience, Inc. (Seattle, WA, USA) carried out genetic engineering using *Spirulina* and expressed the single-chain antibody fragments targeting *Campylobacter*. The biopharmaceutical protein expressed by *Spirulina* accounted for up to 15% of the total biomass. No purification was needed for the proteins produced, and the proteins were stable without refrigeration. Mice tests have demonstrated a preventive effect against the disease through oral administration. The product has now been advanced to phase 1 clinical trials in humans [112].

## 6. Remaining Challenges

Synthetic biology has been regarded as one of the most important biotechnological platforms in the 21st century. Green, economical, and sustainable bio-manufacturing are becoming a promising strategic emerging industry. Great progress has been made in developing cyanobacteria as green microbial cell factories for the sustainable production of valuable natural products, chemicals, and biofuels. However, there are still some challenges in further development.

The major limitation is the lack of refined gene regulatory elements and enabling technologies. Although the “synthetic biology toolkit” for cyanobacteria metabolic engineering is developing rapidly, there is still a lag behind the commonly used industrial strains, such as *E. coli* and yeasts, which resulted in low yields of the target products made by cyanobacteria. Studies on the development of efficient gene regulatory elements allow the optimization of photosynthetic yield and carbon flux and thus may help achieve the full potential of cyanobacterial cell factories.

The low adaptability of standardized gene assembly modules in cyanobacteria is also a problem. The development and optimization of appropriate gene-assembly modules for cyanobacteria, such as the Syne Brick vectors and the CyanoGate modular cloning system, will largely accelerate the production efficiency of cyanobacterial cell factories [113].

Another challenge is the genetic instability of the engineering strains of cyanobacteria. Achieving the specificity and tunability of introduced genes and pathways is another large hurdle for metabolic engineering in cyanobacteria. This can minimize the loss or uncontrolled expression of heterologous genes and some other negative effects on modified cells [114,115].

Currently, most cyanobacterial cultures still require closed systems as they may not be able to grow in a highly selective environment resulting from contamination. The high cost thus limits the large-scale industrial application of cyanobacteria. There are other problems in that there is a need to design industrial bioreactors according to the required light and intensity, which could be quite important considering the self-shading of cells in the culture medium [116].

## 7. Future Prospects

Synthetic biological tools and strategies are used in the artificial design and optimization of light-driven carbon-fixing cell factories. Improving the efficiency of photosynthetic carbon fixation has become the most attractive direction of research and development, which fundamentally determines the efficacy and potential of photosynthetic microbial platforms. This includes improving the efficiency of light harvesting, transfer, utilization, and enhancing the activity and stability of the key and rate-limiting enzyme RuBisCO in the Calvin–Benson cycle. Regarding the stability of growth and production in the microalgal industry, we need to focus on the tolerance of algal species, the physiological robustness, and the stress resistance of the chassis algal strains used for genetic engineering to maximize the realization of the potential of synthetic carbon fixation.

In recent years, cyanobacteria has played important roles in the field of cell therapy for the treatment of cardiovascular diseases, breast cancer, chronic diabetes, etc. [117,118]. It is also applied to the bioremediation of saline soil and wastewater with heavy metals. In agricultural production, photosynthetic microorganisms are used as biofertilizers to improve plant growth, yield, and soil bioavailability [119].

Owing to the development of the light-driven synthetic platforms and regulatory tools, and the deepening of the understanding of the metabolic mechanisms, including the photosynthetic carbon fixation and central metabolism, various types of highly efficient cell factories in the synthesis of biofuel products and bio-based chemicals will be further stimulated. The application of the cyanobacterial cell factory and light-driven synthetic biology will to some extent alleviate the adverse impact of CO_2_ on the ecosystem and provide a strong boost to green biomanufacturing and sustainable economic development.

## Figures and Tables

**Table 1:** Common cyanobacterial model organisms.

Host	Delivery Approach	Genome Size	Doubling Time	Optimum Temperature	References
*Synechocystis* sp. PCC6803	Natural transformation	3.6 Mbp	6.6 h	30 °C	[15]
*Synechococcus* sp. PCC7002	Natural transformation/Conjugation	3.01 Mbp	2.27 h	38 °C	[50]
*S. elongatus* PCC7942	Natural transformation	2.7 Mbp	4.9 h	38 °C	[15]
*S. elongatus* UTEX2973	Conjugation	2.7 Mbp	1.93 h	38 °C	[50]
*Anabaena* sp. PCC7120	Conjugation	6.4 Mbp	14 h	23 °C	[36,51]

## Data Availability

Not applicable.
